# Knowledge and Practices of Female Nurses at Primary Health Care Clinics in Gaza Strip-Palestine Regarding Early Detection of Breast Cancer

**DOI:** 10.31557/APJCP.2021.22.11.3679

**Published:** 2021-11

**Authors:** Husam H Mansour, Fatma A Shallouf, Ahmed A Najim, Yasser S Alajerami, Khaled M Abushab

**Affiliations:** 1 *Department of Radiology, Al-Shifa Hospital, Gaza, Palestine Medical Imaging Department, Al-Azhar University, Gaza, Palestine. *; 2 *Ministry of Health, Gaza-Palestine, Palestine. *; 3 *Department of Nursing, Al-Azhar University, Gaza-Palestine, Palestine. *; 4 *Department of Medical Imaging, Al-Azhar University, Gaza-Palestine, Palestine. *

**Keywords:** Breast cancer, knowledge, nurses, practices, Gaza Strip, Palestine

## Abstract

**Background:**

Breast cancer (BC) is the leading cause of cancer deaths among females in Palestine. Female nurses play a vital role in increasing women’s awareness of BC early detection.

**Objective:**

This study aimed to assess the knowledge and practices of female nurses at Primary Health Care Clinics (PHCCs) in the Gaza Strip regarding early detection of BC.

**Materials and Methods:**

This is an analytical, cross-sectional study with a census sample that includes all target female nurses (152) currently working at PHCCs. The study was conducted during the period February 2019 - March 2020. A structured self-administered questionnaire was used to collect data among female nurses. Descriptive and inferential analyses were used to examine the relationship between the variables. Ethical approval was obtained from a Helsinki Committee Gaza Strip-Palestine.

**Results:**

The nurses demonstrated a good knowledge of signs and risk factors of BC, with scores of 85.3% and 77.9%, respectively. The majority of the participants correctly defined breast self-examination (BSE) and claimed that clinical breast examination (CBE) is a useful tool to detect BC (94.1% and 97.4%, respectively). Nurses who had previous training in CBE had better knowledge than those who had not (t = 3.5; P-value <0.001). Nurses who previously performed mammography had a knowledge score (mean ± SD = 78.1±12.8) higher than those who did not (mean ± SD = 72.5±14). Nurses having previous training had a knowledge score of 8.9 times higher than those without relevant training (t = 4.2, P-value < 0.001). Nurses’ knowledge of BC risk factors increased the practicing score by a factor of 0.22 (t = 3.0, P-value = 0.003).

**Conclusion:**

Nurses demonstrate good knowledge and practices of early BC detection. Previous education sessions affect the knowledge of early detection methods positively.

## Introduction

Cancer is one of the leading causes of mortality among adults all over the world. Breast cancer (BC) is the most frequent cancer among women, affecting 2.1 million women each year, resulting in an increasing number of cancer-related deaths among women. In 2018, it was estimated that 627,000 women died from BC, representing approximately 15% of all cancer deaths among women. While BC rates are higher among women in more developed regions, rates are increasing in nearly every region globally (WHO, 2018). BC is the second most commonly diagnosed cancer throughout the world, next to lung cancer. It is the most commonly diagnosed cancer among female subjects (Santhanakrishnan et al., 2016). A long-term increase in the incidence of the disease has been observed in both developed and developing countries. In 2014 BC accounted for 1.03% of the total annual deaths, yielding an age-adjusted death rate of 21.40 per 100,000 of the population (Andegiorgish et al., 2018).

Female nurses play a critical role in increasing women’s awareness of early detection and by providing helpful information about BC. Therefore, it is necessary for them to emphasize that the early detection of the BC by breast self-exam (BSE), clinical breast exam (CBE), and mammography is essential to reduce the morbidity and the mortality associated with this cancer (Bancej et al., 2003). Annual reports illustrate that BC is the most prevalent disease in the Gaza Strip, representing 20.5% of the total cancer patients (MoH, 2017). Thus, it is urgent to organize a series of health education plans and update the knowledge of BC and screening practices. In this effort, female nurses can educate, disseminate and transfer their knowledge to the community. To improve BC outcomes and survival, early detection is critical. All healthcare personnel should have adequate knowledge regarding BC early detection, and they should be role models. Hence, this study aimed to assess the knowledge and practices regarding BC early detection among female nurses at Primary Health Care Clinics (PHCCs) in the Gaza Strip.

## Materials and Methods

*Study design*

The design of the study is analytical; cross-sectional analysis was used to assess the level of the knowledge and practices regarding BC early detection among PHCC female nurses in the Gaza Strip. The sample of this study was a census, which consists of female nurses (152 female nurses) currently working at the PHCCs. A structured self-administered questionnaire was used for data collection.

Instrument of the study

A structured self-administered questionnaire was developed with closed-ended questions. The questionnaire has six parts: (1) demographic characteristics and medical history, such as age, qualifications, years of experience, and whether anyone in your family has been diagnosed with BC; (2) knowledge of risk factors of BC. (3) knowledge of early BC detection; (4) knowledge regarding the practice of BSE; (5) knowledge and practice of CBE, and (6) knowledge and practice of Mammography.

The questionnaire was constructed after reviewing the literature and was evaluated by a panel of experts in the field of Mother and Child Health (MCH) and research methodology. The reliability of an instrument is the degree of consistency of the questionnaire. For this purpose, the reliability coefficient for the study was measured. The Cronbach’s coefficient alpha result was 0.717, which is considered an acceptable reliability.

*Statistical analysis*

The Statistical Package for Social Science (SPSS version 23) was used for data entry and analysis. The continuous variables were presented as the mean ± standard deviation (SD) and categorical variables as number and percentage. Inferential analysis, including Pearson correlation, t-test and multiple linear regression to predict factors affecting participants’ knowledge about early BC detection and the practice of BSE. The confidence interval was considered at 95%. A p-value ≤ 0.05 is statistically significant.

A score based on breast cancer early detection methods was calculated by the summation of thirteen questions. Every correct answer was scored one point, then the figure was multiplied by 100 and divided by 13. Scores regarding BSE were calculated by the summation of eight questions. Knowledge of risk factors was assessed by answers to 10 questions, and knowledge about signs and symptoms was determined by answers to 11 questions. Eight questions were used to assess the practice of BSE. Scores for knowledge of early BC detection and practices were categorized as Poor < (60%), Fair (60-80%), and Good (>80%).

## Results

*Socio-demographic characteristics of study nurses*

More than half of the study participants were within the age group 35-45 years. The participants’ qualifications were classified as having a diploma, a bachelor’s degree, and a master’s degree in percentages of 42.8%, 52.6%, and 4.6%, respectively. Approximately 46.7% had experience of 15 years or less. One-fifth of study participants had a family history of BC.

*Knowledge of signs and risk factors of BC*

A good knowledge score was found regarding knowledge of signs (85.3%) and risk factors (77.9%) of BC. Table 1 presents the participants’ responses to BC risk factors. The well-known BC risk factors are family history (98.7%), hormonal replacement therapy (92.8%), contraceptive usage (86.8%), nulliparous (86.2%), and age (81.6%). Low scored risk factors in the current study included stress, late menopause, age at first pregnancy, early age at menarche, and overweight. The overall response score for the risk factors of BC is 77.9%.

*Responses of participants regarding signs and symptoms of BC*

Table 2 shows participants’ knowledge of the signs and symptoms of BC. The results reveal an excellent knowledge regarding the change in the size of the breast, swelling or enlarged lymph nodes in axillae, lump under the armpit, changes in the shape of the breast, lump in the breast, and discharge, as all the percentages were above 90%.

*Knowledge of the practice of BSE*

The majority of the participants correctly defined BSE and knew thee frequency at which it is done in percentages of 94.1%, 81.6%, respectively. Only 44.1% knew the time at which women should start doing BSE. More than half knew that BSE involves inspection of the shape and size of the breast. The majority of the participants are practicing BSE (86.2%).

*Knowledge and performing CBE*

Approximately half of the participants (48%) had previous training about performing CBE. However, the majority (97.4%) claimed that CBE is a useful tool to detect BC, and knew that it is done using the hand (84.2%). They answered that a doctor, a trained nurse, or a trained midwife performs CBE (27.8%, 32.8%, 26.8%, respectively). Only 12.6% of participants answered incorrectly, stating that it is done by the individual herself.

*Knowledge and practice of mammography*

Regarding the participants’ knowledge of mammography, the majority of the participants considered mammography a useful tool for the early detection of BC (92.7%). Roughly 90% knew the best age to perform mammography. 79.6% answered that mammography is performed yearly, and 14.5% said that it is done when a lump is found on BSE or CBE. The participants of the current study gave explanations about not performing mammography. The most recognized explanations were; not old enough to do mammography (37%), do not have a breast problem (17%), mammography is not available (8%), fear of results (8%), financial constraints (8%) and not thinking about it (8%). Other reasons were recognized among a few participants (10%). 

*Relationship between knowledge and practice*

There is a positive relationship between practicing BSE and the other three variables related to knowledge (knowledge about BC early detection methods, knowledge about risk factors, and knowledge about signs and symptoms). A relationship was shown between participants’ early BC detection methods and practicing BSE. The Pearson correlation test showed a weak positive statistically significant relationship (r = 0.274, P-value = 0.001). A significant moderate positive correlation was found between practice score and knowledge of BC risk factors and signs and symptoms (r = 0.339, 0.236; P-values <0.001, P-values = 0.003), respectively. Another statistically significant correlation was between knowledge of BC risk factors, and knowledge of breast signs and symptoms (r = 0.463, P-value <0.001). Finally, the study did not reveal a statistically significant correlation between participants’ knowledge of early BC detection methods and each of the risk factors and BC signs and symptoms (P-values > 0.05).

*Differences between knowledge scores and some variables*

Results demonstrate that the presence of family history does not affect the knowledge of BC, (t= 0.337, P-value = 0.150). Employees who had previous training about BCE have better knowledge (mean ± SD = 77.5±12.6) than those who have not (mean ± SD = 69.7±14.1), and the difference is statistically significant (t = 3.5; P-value <0.001). Nurses that previously performed mammography have a knowledge score (mean ± SD = 78.1±12.8) greater than those who did not (mean ± SD = 72.5±14), however, the difference is not statistically significant (t= 1.8; P-value= 0.06).

Results show that there are no statistically significant differences between practicing BSE among the study participants with respect to two factors: presence of family history or previous training in CBE (P-value > 0.05). A statistically significant difference was seen between participants’ practicing BSE and performing mammography (t= 3.0, P-value = 0.004).

*Factors affecting participants about early BC detection methods*

Table 3 shows multiple linear regression analysis based on a model consisting of six important variables that have a significant effect on participants’ knowledge about early BC detection methods (B= 49.9, P-value < 0.001). The test reveals two predictors affecting the knowledge score: previous education sessions and knowledge of BC signs and symptoms. Previous education sessions affect knowledge of early BC detection methods positively. Those with previous training have a knowledge score 8.9 times greater than those who have not, holding other variables constant (t= 4.2, P-value < 0.001). A one-unit increase in the practice of BSE will increase knowledge by a factor of 5.4, holding other variables constant (t= 2.11, P-value= 0.036).

*Factors affecting participants’ practice BSE*

Table 4 shows multiple linear regression analysis. The model consists of five important variables that have a significant effect on the participants’ practice score of BSE (B = 30.4, P-value = 0.001). The test reveals two predictors affecting the practice score: knowledge about BC risk factors will increase the practicing score by a factor of 0.22 holding other variables constant (t = 3.0, P-value= 0.003); the knowledge score affects the practice positively in that a one-unit increase in knowledge score will increase the practice score by a factor of 0.28 holding other variables constant (t = 2.66, P-value = 0.008). 

**Table 1:** Responses of Participants about Risk Factors of BC

Factors	Yes	No	Mean%
The family antecedent of BC (Hereditary)	150 (98.7)	2 (1.3)	98.7
Hormone replacement therapy (HRT) for menopause	141 (92.8)	11 (7.2)	92.8
Use of oral contraceptives	132 (86.8)	20 (13.2)	86.8
Nulliparous	131 (86.2)	21 (13.8)	86.2
Risk increases with age	124 (81.6)	28 (18.4)	81.6
Stress	112 (73.7)	40 (26.3)	73.7
Late menopause (age ≥ 55 years)	105 (69.1)	47 (30.9)	69.1
Age at first pregnancy ≥ 30 years	102 (67.1)	50 (32.9)	67.1
Early age at menarche (≤ 12 years)	96 (63.2)	56 (36.8)	63.2
Overweight	91 (59.9)	61 (40.1)	59.9
Total response			77.91

**Table 2:** Responses of Participants about Signs and Symptoms of BC

Signs and symptoms	Yes	No	Mean%
Change in the size of the breast	147 (96.7)	5 (3.3)	96.7
Swelling or enlarged lymph nodes in the axilla	146 (96.1)	6 (3.9)	96.1
Lump under armpit	146 (96.1)	6 (3.9)	96.1
Changes in the shape of the breast	144 (94.7)	8 (5.3)	94.7
Lump in the breast	141 (92.8)	11 (7.2)	92.8
Discharge from the breast	140 (92.1)	12 (7.9)	92.1
Discoloration/dimpling of the breast	131 (86.2)	21 (13.8)	86.2
Ulceration of the breast	119 (78.3)	33 (21.7)	78.3
Inversion/ pulling in of nipple	110 (72.4)	42 (27.6)	72.4
Scaling / dry skin on nipple region	109 (71.7)	42 (27.6)	72.2
Soreness in the breast	93 (61.2)	59 (38.8)	61.2
Total response			85.3

**Table 3:** Factors Affecting Participants about BC Early Detection Methods

Variable	B	T	P-value	CI	
				UB	LB
(Constant)	49.985	7.704	<0.001*	37.161	62.809
Do you practice BSE?	5.432	1.753	0.082	-0.693	11.557
Did you get any education sessions about CBE before?	8.938	4.234	<0.001*	4.765	13.11
Knowledge about BC signs and symptoms	0.148	2.116	0.036	0.01	0.286
Knowledge about BC risk factors	0.052	0.93	0.354	-0.059	0.163
Department	-1.091	-1.391	0.166	-2.641	0.459
Have you ever done mammography?	3.996	1.433	0.154	-1.514	9.506

*Statistically significant at P ≤ 0.05

**Table 4:** Factors Affecting Participants’ Ppractice BSE

Variable	B	T	P-value	CI	
				UB	LB
(Constant)	30.492	3.303	0.001*	12.247	48.737
Did you get any education session about CBE?	1.138	0.39	0.697	-4.626	6.902
Knowledge about BC signs and symptoms	0.061	0.663	0.508	-0.121	0.243
Knowledge about BC risk factors	0.222	3.035	0.003*	0.077	0.366
Have you ever done mammography?	6.572	1.803	0.073	-0.632	13.775
Knowledge about BC early detection methods	0.285	2.669	0.008*	0.074	0.496

*Statistically significant at P ≤ 0.05

## Discussion

Nurses play an important role within the healthcare system. In most cases, female healthcare providers are the preferred source of information about BC because women feel more comfortable asking them questions. Typically, their work is in the areas of health education and counselling (Alkhasawneh, 2007). Empowering nurses with information about BC, early detection methods, and their related benefits could help in advancing their skills in performing BSE and expanding their role as client educators. Education and awareness need to be culturally appropriate and targeted towards the relevant population, because this may contribute towards an increased early presentation so that the highest benefit can be gained. Health care providers, especially those who come in regular contact with women, can play an important role in providing information regarding BC. Regarding the participants’ responses to BC risk factors, the scores on these subjects was much higher than those reported by Ahmad et al. (2011), who found knowledge scores of most risk factors no greater than 61%. In another study (Andegiorgish et al., 2018), risk factor scores were less than 68%. 

Consistent with an overall response score of 77.9 on the risk factors of breast cancer, Yousuf et al., (2012) reported that more than 75% of Saudi nurses knew more than 50% of the questions related to risk factors. Participating Jordanian nurses demonstrated good knowledge related to risk factors of BC (Suleiman, 2014). In contrast, Taranikanti et al., (2014) reported that knowledge of BC risk factors among nurses in their study was poor, with scores of less than 50% except for the use of contraceptives. Similar results were presented in other studies that showed unsatisfactory knowledge of BC risk factors, with a mean score of less than 50% (Ghanem et al., 2011; Fotedar et al., 2013). Other studies reporting low knowledge among nurses of the risk factors of BC include those of Yousuf et al., (2012) and Lemlem et al., (2013). while another study has reported nurses’ knowledge of risk factors of BC to be high (Awodele et al., 2009). There is consensus that knowledge of BC risk factors is essential to aid nurses and midwives in giving appropriate counselling and recommendations to their patients. This is especially true for those at high risk of developing breast cancer. The absence of a well-organized BC screening program in the GS makes this knowledge even more important.

Regarding excellent knowledge (90%) of the signs and symptoms of breast cancer, our results are consistent with another study regarding a lump in the breast, as 93.6% responded correctly (Odusanya et al., 2001). Good knowledge is also shown regarding other BC signs and symptoms. A study conducted by Benaicha et al., (2016) revealed that nurses have good knowledge of BC signs and symptoms. The majority of our participants are trained well about BC and related risk factors. There is an international interest and interest from the MoH in the Gaza Strip in educating the nurses about signs and symptoms of BC and methods of early detection, especially in PHC centers, as they are the first line in early BC detection. Frequent campaigns and awareness about early detection methods may affect their knowledge about the disease and its related risk factors, signs, and symptoms.

The majority of the study participants are practicing BSE (86.2). This result is much higher than that reported in a study published by Sani & Yau (2018), which showed that the practice of BSE among health workers was only 65.3%. Nguefack et al. (2017) reported this percentage to be 77.5%. About 94% of nurses in Singapore reported practicing BSE (Chong et al., 2015), 90.6% by Zeru et al., (2019), 70.4% among Turkish nurses (Erbil and Bolukbas, 2014), and 50% in Malesia (Khokher et al., 2015). These results and ours are higher than that of another study reported at 21.4% by Getu et al., (2018). Despite the participants’ knowledge about the steps of performing BSE, they are not performing it regularly. The gap between their awareness of the steps of practicing BSE and not practicing was previously reported, and the women in the literature gave some reasons for it. These include carelessness, forgetfulness, fear of being diagnosed with BC, discomfort, and lack of privacy (Asiri & Rashad, 2019; Shallo & Boru, 2019; Alomair et al., 2020). It has been previously reported by Akpınar et al., (2011) that health care professionals practice BSE regularly at low (27.3%) percentages. 

The literature shows that BC could be detected more accurately when CBE is performed adjacent to mammography as a screening tool (Provencher et al., 2016). Health professionals can be taught successfully to improve their CBE accuracy and skills (Pace et al., 2018). There is a need for continuing professional education programs for nurses, as they are always involved in patient care and education. We advise that nurses should systematically conduct CBE of their patients, especially in developing countries like Palestine, where screening programs by mammography are not well organized.

Regarding the participants’ knowledge of mammography, our results are in line with another study conducted in Palestine, which showed that barriers to conducting screening mammography among health care providers include being busy (46.7%), do not think the patient has BC (41.5%), the patient does not have symptoms (31.9%), and do not want to know whether the patient has BC or not (26.7%). Other reasons identified by a study conducted by Nazzal et al., (2018) include shyness about exposing breasts, mammography is painful, and mammography causes adverse effects. The literature shows a reduction of mortality from BC by 25 to 30% using mammograms (Marmot et al., 2013).

Consistent with our findings of early BC detection methods and practicing BSE, a weak significant positive relationship (r=0.242, P = 0.001) was shown between the knowledge and the practice in a study by Sani and Yau (2018). Alazmi et al., (2013) reported a significant positive correlation between knowledge of BC and practicing BSE. Their result showed a significant positive correlation between knowledge of BC and practicing BSE. They believed that this correlation could be related to training and education sessions about CBE. Women who had adequate knowledge of BSE were more likely to practice it than women who have not.

In conclusion, the current study is important to assess the knowledge and practices of early BC detection among female nurses. The study revealed a good knowledge of BC signs, risk factors, and early detection methods. Nurses have good practices regarding early detection methods of BC. The nurses got the opportunity of training in BC screening methods and educational campaigns regarding early detection of BC. This provides them with a better awareness of how to perform BSE. Nurses should be knowledgeable and aware of the early detection of BC by BSE, CBE, and mammography. Also, nurses should be encouraged to have ready access to reliable, up-to-date information and continuing courses programs concerning early BC detection. Future research should study the reason for health care provider attitudes regarding noncompliance with performing mammography.

## Author Contribution Statement

Shallouf FA., Mansour HH. and Najem AA. participated in idea formation, data gathering. Alajerami YS and Abushab KM participated in data analysis and interpretation. All contributors participated in manuscript drafting, revising and approved the manuscript and agreed with study publication.

## Consent for Publication

This paper is based on the master’s dissertation of Fatma A. Shallouf in 2020 from Maternal and Child Health at Al-Quds university, Gaza-Palestine.

## Ethical Statement

Ethical approval was obtained from a Helsinki Committee (Number: PHRC/HC/609/19). All willing participants signed a written consent form with the assurance of confidentiality of obtaining data. 

## Availability of Supporting Data

The data used to support the findings of this study are available from the corresponding author upon request.

## Conflict of Interest 

The authors declare no conflict of interest concerning the research

## References

[B1] Ahmad S, Qureshi AN, Atta S (2011). Knowledge, attitude and practice for breast cancer risk factors and screening modalities in staff nurses of Ayub Teaching Hospital Abbottabad. J Ayub Med College.

[B2] Akpinar YY, Baykan Z, Naçar M, Gün I, Çetinkaya F (2011). Knowledge, attitude about breast cancer and practice of breast cancer screening among female health care professionals: a study from Turkey. Asian Pac J Cancer Prev.

[B3] Alazmi SF, Alkhabbaz A, Almutawa HA (2013). Practicing breast self-examination among women attending primary health care in Kuwait. Alexandria J Med.

[B4] Alkhasawneh IM (2007). Knowledge and practice of breast cancer screening among Jordanian nurses. Oncol Nurs Forum.

[B5] Alomair A, Felemban D, Felemban M (2020). Knowledge, attitude, and practice of breast self-examination toward breast cancer among female students at King Saud University in Riyadh, Saudi Arabia. IJMDC.

[B6] Andegiorgish AK, Kidane EA, Gebrezgi MT (2018). Knowledge, attitude, and practice of breast cancer among nurses in hospitals in Asmara, Eritrea. BMC Nurs.

[B7] Asiri H, Rashad W (2019). Prediction of the awareness and practice of breast self–examination among females using health Believe Model. A literature reviews. IOSR-JNHS.

[B8] Awodele O, Adeyomoye AA, Oreagba IA (2009). Knowledge, attitude and practice of breast cancer screening among nurses in Lagos University Teaching Hospital, Lagos Nigeria. Nig Q J Hosp Med.

[B9] Bancej C, Decker K, Chiarelli A (2003). Original paper: Contribution of clinical breast examination to mammography screening in the early detection of breast cancer. J Med Screen.

[B10] Chong P, Krishnan M, Hong C, Swah T (2015). Knowledge and practice of breast cancer screening amongst public health nurses in Singapore. Singapore Med J.

[B11] Erbil N, Bolukbas N (2014). Health beliefs and breast self-examination among female University nursing students in Turkey. Asian Pac J Cancer Prev.

[B12] Fotedar V, Seam RK, Gupta MK (2013). Knowledge of risk factors & early detection methods and practices towards breast cancer among nurses in Indira Gandhi medical college, Shimla, Himachal Pradesh, India. Asian Pac J Cancer Prev.

[B13] Getu MA, Wudu Kassaw M, Tlaye K, Gebrekiristos AF (2018). Assessment of breast self-examination practice and its associated factors among female undergraduate students in Addis Ababa University, Addis Ababa, Ethiopia, 2016. Breast Cancer Targets Ther.

[B14] Ghanem S, Glaoui M, Elkhoyaali S (2011). Knowledge of risk factors, beliefs and practices of female healthcare professionals towards breast cancer, Morocco. Pan Afr Med J.

[B15] Khokher S, Qureshi MU, Fatima W, Mahmood S, Saleem A (2015). Impact of a breast health awareness activity on the knowledge level of the participants and its association with socio-demographic features. Asian Pac J Cancer Prev.

[B16] Lemlem SB, Sinishaw W, Hailu M, Abebe M, Aregay A (2013). Assessment of knowledge of breast cancer and screening methods among nurses in University hospitals in Addis Ababa, Ethiopia, 2011. ISRN Oncol.

[B17] Marmot MG, Altman DG, Cameron DA (2013). The benefits and harms of breast cancer screening: An independent review. Br J Cancer.

[B19] Muhammad Sani A, Labaran Yau S (2018). Relationship between knowledge and practice of breast self-examination among female workers in Sokoto, Nigeria. Int J Gynaecol Obstet.

[B20] Nazzal Z, Sholi H, Sholi SB, Sholi MB, Lahaseh R (2018). Motivators and barriers to mammography screening uptake by female health-care workers in primary health-care centres: A cross-sectional study. Lancet.

[B21] Nguefack CT, N’djeudjui C, Engbang JP (2017). Knowledge, attitude, and practice on breast cancer among health professionals in Douala references hospitals, Cameroon. J Cancer Educ.

[B22] Odusanya OO, Tayo OO (2001). Breast cancer knowledge, attitudes and practice among nurses in Lagos, Nigeria. Acta Oncol.

[B23] Pace LE, Dusengimana JV, Keating NL (2018). Impact of breast cancer early detection training on Rwandan health workers’ knowledge and skills. J Glob Oncol.

[B24] Provencher L, Hogue J, Desbiens C (2016). Is clinical breast examination important for breast cancer detection?. Curr Oncol.

[B25] Santhanakrishnan N, Prabakaran S, Singh Z (2016). Knowledge, attitude, and practice regarding breast cancer and its screening methods among nursing staff working in a tertiary-care hospital located in South India. Int J Med Sci Public Health.

[B26] Shallo SA, Boru JD (2019). Breast self-examination practice and associated factors among female healthcare workers in western Ethiopia 2019: A cross-sectional study. BMC Res. Notes.

[B27] Suleiman A (2014). Awareness and attitudes regarding breast cancer and breast self-examination among female Jordanian students. J Basic Clin Pharm.

[B28] Taranikanti M, Panda S, Dash A (2014). Knowledge of nurses about breast cancer risk factors, general awareness and screening procedures in South India. Int J Med Sci Public Health.

[B30] Yousuf SA, Al Amoudi SM, Nicolas W, Banjar HE, Salem SM (2012). Do Saudi nurses in primary health care centres have breast cancer knowledge to promote breast cancer awareness?. Asian Pac J Cancer Prev.

[B31] Zeru Y, Sena L, Shaweno T (2019). Knowledge, Attitude, practice, and associated factors of breast cancer self-examination among urban Health extension workers in Addis Ababa, Central Ethiopia. J Midwifery Womens Health.

